# Academic social comparison orientation and adaptive academic help-seeking among university students: academic anxiety as a mediator and academic self-efficacy as a moderator

**DOI:** 10.3389/fpsyg.2026.1872436

**Published:** 2026-09-21

**Authors:** Bandar Salah Almailabi

**Affiliations:** Department of Education, Islamic University of Madinah, Madinah, Saudi Arabia

**Keywords:** academic anxiety, academic self-efficacy, academic social comparison orientation, adaptive academic help-seeking, university students

## Abstract

**Introduction:**

Academic help-seeking is a self-regulatory academic behavior that may be shaped by students’ evaluative experiences and beliefs about their academic capability. This study examined whether academic anxiety statistically mediates the association between academic social comparison orientation and adaptive academic help-seeking, and whether academic self-efficacy moderates the association between academic anxiety and adaptive academic help-seeking among university students.

**Methods:**

A cross-sectional online survey was conducted with 218 undergraduate students from two Saudi universities. The main model was analyzed using partial least squares structural equation modeling (PLS-SEM) with 5,000 bootstrap resamples.

**Results:**

Academic social comparison orientation was positively associated with academic anxiety (β = 0.374, *p* < 0.001), and academic anxiety was negatively associated with adaptive academic help-seeking (β = −0.234, *p* = 0.001). Academic social comparison orientation also showed a negative direct association with adaptive academic help-seeking (β = −0.192, *p* = 0.004). The indirect effect through academic anxiety was negative and statistically significant (β = −0.087, *p* = 0.005), consistent with partial statistical mediation. Academic self-efficacy moderated the anxiety–help-seeking association (β = 0.153, *p* = 0.010), with the positive interaction indicating that the negative association between academic anxiety and adaptive academic help-seeking became weaker as academic self-efficacy increased.

**Conclusion:**

The findings suggest that academic anxiety partially statistically mediated the association between stronger academic social comparison orientation and lower adaptive academic help-seeking, whereas academic self-efficacy statistically moderated the negative association between academic anxiety and adaptive academic help-seeking, such that this association was weaker at higher levels of academic self-efficacy. Given the cross-sectional design, the findings should be interpreted as statistical associations consistent with the proposed theoretical model rather than as evidence of causal effects.

## 1 Introduction

Academic help-seeking has been described in the higher education literature not simply as a reaction to academic difficulty, but also as part of students’ self-regulated academic functioning. Li et al. (2023) characterize academic help-seeking as an important self-regulation strategy for college students and organize the literature around its definition, its relationship with academic performance, the resources through which students seek help, and the factors associated with it. Meta-analytic evidence among postsecondary students further indicates that need-contingent help-seeking shows a very small positive association with achievement, whereas the type and source of help-seeking matter: instrumental and formal help-seeking are positively associated with academic performance, whereas avoidant and executive help-seeking are negatively associated with achievement (Fong et al., 2023). Similarly, Karabenick and Knapp (1991) reported that, in academic contexts, help-seeking was positively related to instrumental achievement activities and to the use of cognitive, metacognitive, and resource-management learning strategies, concluding that the evidence was more consistent with viewing help-seeking as an achievement-related behavior than as a dependent behavior. Taken together, this literature positions adaptive help-seeking as a strategic academic behavior. At the same time, because asking for help often involves acknowledging uncertainty or difficulty to peers or instructors, help-seeking decisions may be shaped by the evaluative conditions students experience in academic settings.

From the perspective of social comparison theory, individuals evaluate their abilities and opinions partly by comparing themselves with others. In Festinger’s (1954) classic formulation, people are motivated to evaluate their opinions and abilities, and when objective standards are unavailable or insufficient, they often rely on comparisons with relevant others. This general tendency has been operationalized through the Iowa–Netherlands Comparison Orientation Measure (INCOM), which assesses individual differences in social comparison orientation, particularly the tendency to compare one’s abilities and opinions with those of others (Gibbons and Buunk, 1999). In academic settings, this tendency may appear as academic social comparison orientation (ASCO), which refers to students’ tendency to compare their academic abilities and academic opinions with those of their peers. In the present study, ASCO is treated as a comparison orientation rather than as an indicator of perceived academic superiority or inferiority.

Academic anxiety refers to anxiety experienced in relation to academic tasks, achievement demands, and academic evaluation. In higher education, academic anxiety has been discussed as a context-specific form of anxiety that occurs within academic settings and differs from broader categories such as state or trait anxiety, although it may take multiple forms, including test anxiety, language learning anxiety, mathematics anxiety, and public speaking anxiety (Idoiaga-Mondragon et al., 2025). Bülbül and Odacı (2023) similarly define academic anxiety as worry, irritation, and nervous feelings related to academic tasks and achievement and describe it as involving cognitive, affective, and behavioral components. For the present model, the evaluative dimension of academic anxiety is especially relevant because adaptive help-seeking may require students to disclose academic difficulty in contexts where their competence could be judged by peers or instructors.

Direct evidence linking ASCO to academic anxiety remains limited. However, broader research suggests that comparison-based self-evaluations may be relevant to anxiety-related outcomes. A systematic review and meta-analysis found that less favorable social comparison evaluations are associated with higher anxiety-related outcomes (McCarthy and Morina, 2020). Experience-sampling evidence also suggests that social anxiety is associated with less favorable and more unstable social comparisons in daily life, including among undergraduate participants (Goodman et al., 2021). More proximal evidence from college science courses indicates that academic social comparison is related to fear of negative evaluation, an anxiety-relevant evaluative concern, although this work conceptualized academic social comparison as students’ perceived standing relative to peers rather than as a comparison orientation measured through an INCOM-based framework (Pigart et al., 2024). These findings do not directly test ASCO as defined in the present study, but they support the expectation that students who are more strongly oriented toward academic comparison may be more likely to experience academic contexts through peer-referenced evaluation. On this basis, higher ASCO was expected to be positively associated with academic anxiety.

Academic anxiety may also be relevant to adaptive academic help-seeking because asking for assistance can be experienced as evaluatively costly when students believe that it exposes their difficulty or limited competence. Research on help-seeking attitudes has shown that perceived threat and perceived benefits are important for understanding students’ avoidance of help-seeking, whereas perceived benefits are more directly related to adaptive help-seeking (Ryan and Pintrich, 1997). Similarly, evidence from mathematics classrooms indicates that mastery orientation is associated with lower mathematics anxiety and greater help-seeking, whereas performance-avoidance orientation predicts higher mathematics anxiety and lower help-seeking behavior; mathematics anxiety itself is also negatively correlated with help-seeking behavior (Federici et al., 2015). Although this evidence comes primarily from school-based and mathematics contexts rather than from broad university academic settings, it supports the expectation that academic anxiety may be negatively associated with adaptive academic help-seeking.

These strands of evidence point to a possible indirect pathway in which stronger academic comparison orientation may be associated with greater academic anxiety, which in turn may correspond to lower adaptive academic help-seeking. Direct evidence testing academic anxiety as a mediator in the association between ASCO and adaptive academic help-seeking remains limited. Nevertheless, the proposed indirect pathway is theoretically plausible because comparison-based academic self-evaluations may heighten anxiety-relevant evaluative concerns, and such concerns may reduce students’ willingness to seek help when doing so could reveal difficulty or limited competence. Thus, academic anxiety was examined as a possible statistical mediator linking ASCO to adaptive academic help-seeking.

Academic self-efficacy refers to students’ judgments of their ability to organize and execute academic tasks under specific task conditions. Within social-cognitive theory, such efficacy beliefs are treated as context-sensitive determinants of students’ effort, persistence, and emotional reactions in academic settings (Pajares, 1996). In higher education, prior studies have reported negative associations between academic self-efficacy and academic anxiety or anxiety-related symptoms, suggesting that students who feel more capable of managing academic demands tend to report lower anxiety (Gutiérrez García and Landeros Velazquez, 2020; Purwanti et al., 2020). Academic self-efficacy is also relevant to help-seeking: among undergraduate students, higher academic self-efficacy has been associated with more instrumental help-seeking and less executive or avoidant help-seeking (Xie and Xie, 2019). Although these associations do not directly establish a moderating effect, they provide an empirical basis for examining the proposed moderating role of academic self-efficacy.

Academic self-efficacy may condition the association between academic anxiety and adaptive academic help-seeking because students with stronger efficacy beliefs may be more likely to view academic difficulty as manageable and to interpret help-seeking as a strategic learning response rather than as evidence of incompetence. Consistent with this rationale, recent evidence indicates that academic self-efficacy buffers the negative effect of help-seeking threat on students’ help-seeking behaviors, although this evidence was obtained from middle-school students and focused on help-seeking threat rather than academic anxiety in higher education (Yang et al., 2025). Therefore, academic self-efficacy was examined as a moderator of the association between academic anxiety and adaptive academic help-seeking, with the expectation that this negative association would be weaker at higher levels of academic self-efficacy.

Across school-based and university research, help-seeking has a social and potentially evaluative dimension, with perceived threat particularly relevant to help-seeking avoidance (Ryan and Pintrich, 1997; Karabenick, 2004). However, some closely relevant prior evidence has been obtained from younger students and subject-specific settings, including middle-school mathematics samples (Ryan and Pintrich, 1997; Federici et al., 2015). University settings differ in important respects from these contexts: college students typically navigate their academic environment more independently (Fong et al., 2023), while large university classes may provide less sustained contact and interpersonal support and present additional psychological and logistical barriers to seeking assistance (Karabenick, 2004). Because academic help-seeking is an important self-regulatory strategy in postsecondary education (Li et al., 2023; Fong et al., 2023), these contextual differences provide a basis for examining the proposed relationships directly among university students rather than assuming that findings from younger or subject-specific samples generalize directly to higher education.

### 1.1 The present study

The reviewed literature provides a theoretical and empirical basis for linking academic social comparison orientation, academic anxiety, academic self-efficacy, and adaptive academic help-seeking. However, limited evidence has examined these constructs within an integrated model of university students that treats academic anxiety as a statistical mediator between academic social comparison orientation and adaptive academic help-seeking, and academic self-efficacy as a moderator of the anxiety–help-seeking association. This gap is important because comparison-oriented academic self-evaluation may be associated with greater academic anxiety, whereas students’ efficacy beliefs may condition the strength of the association between anxiety and adaptive help-seeking. To address this gap, the present study tested an integrated statistical model in which ASCO was expected to be positively associated with academic anxiety, academic anxiety was expected to be negatively associated with adaptive academic help-seeking, academic anxiety was evaluated as a statistical mediator, and academic self-efficacy was evaluated as a moderator of the anxiety–help-seeking association. Figure 1 presents the hypothesized research model.

**Figure 1:**
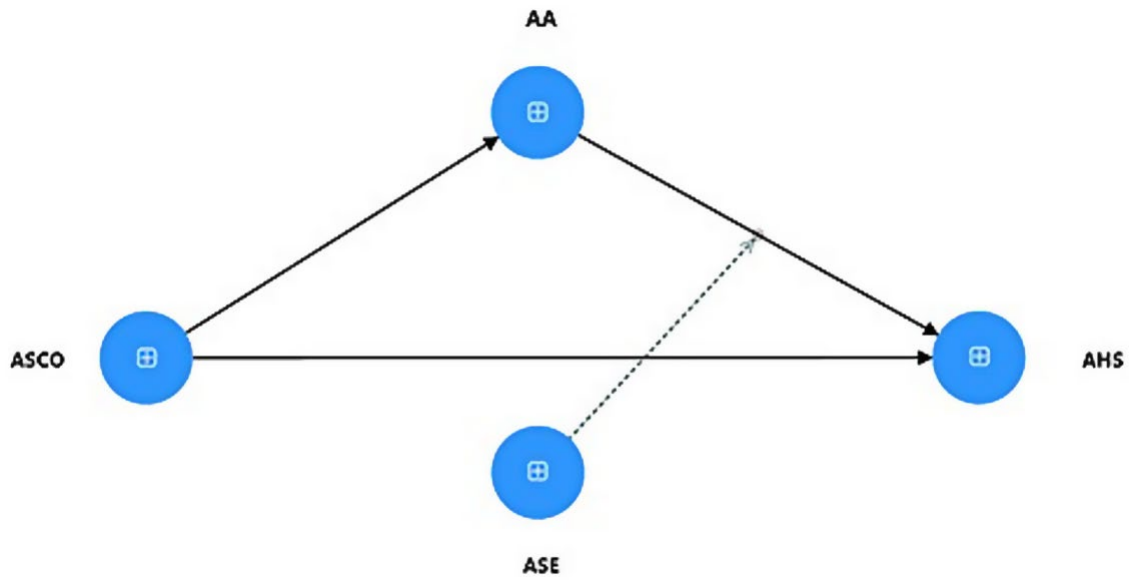
Hypothesized research model. ASCO, academic social comparison orientation; AA, academic anxiety; ASE, academic self-efficacy; AHS, adaptive academic help-seeking. The model specifies academic anxiety as a statistical mediator of the association between ASCO and AHS, and academic self-efficacy as a moderator of the association between AA and AHS.

Accordingly, the following hypotheses were tested:

*H1*: ASCO will be positively associated with academic anxiety.

*H2*: Academic anxiety will be negatively associated with adaptive academic help-seeking.

*H3*: Academic anxiety will statistically mediate the association between ASCO and adaptive academic help-seeking, such that higher ASCO will be associated with higher academic anxiety, which in turn will be associated with lower adaptive academic help-seeking.

*H4*: Academic self-efficacy will moderate the association between academic anxiety and adaptive academic help-seeking, such that the negative association will be weaker at higher levels of academic self-efficacy.

## 2 Methods

### 2.1 Participants and procedure

The study employed a cross-sectional online survey design. Undergraduate students were recruited from the Islamic University of Madinah and Taibah University through official student communication channels. The study therefore used a voluntary, nonprobability sampling approach. Eligible participants were undergraduate students who were currently enrolled at one of the two universities, were able to read and understand the language of the questionnaire, and provided informed consent before participation. Data were collected over a two-week period from April 8 to April 22, 2026. A total of 245 responses were initially received. Responses were excluded if informed consent was not provided, if the questionnaire was incomplete, or if the response was flagged during data-quality screening as clearly indicating a nonserious response, defined as a clearly invariant or patterned response across questionnaire items. The final analytic sample consisted of 218 undergraduate students.

### 2.2 Ethical consideration

The study received ethical approval from the Research Integrity and Ethics Committee at the Islamic University of Madinah (Approval No. 9/12). Electronic informed consent was obtained from all participants before they began the questionnaire. The introductory section of the questionnaire explained that participation was voluntary and that participants could withdraw at any time without penalty. To protect confidentiality, no names or other direct identifying information was collected, and responses were collected anonymously and used solely for research purposes. No incentives were offered for participation.

### 2.3 Instruments

All study instruments were presented to participants in Arabic. The Arabic INCOM short form was already available in a validated Arabic version (Almailabi and Buunk, 2026) and therefore did not require translation in the present study; only its item wording was adapted to the academic context. The other three measures—the Academic Anxiety Scale (AAS), General Academic Self-Efficacy Scale (GASE), and adaptive academic help-seeking measure—were translated from English into Arabic for the present study using a multistep procedure involving forward translation, bilingual review, and preliminary comprehensibility checking. The initial Arabic translations were reviewed by two bilingual specialists whose first language was English and who held doctoral degrees in Arabic language. The specialists reviewed the Arabic wording against the original English items to identify wording discrepancies and assess whether the intended meaning of each item had been retained. A preliminary check was subsequently conducted with eight undergraduate students from the target population to assess item comprehensibility and identify unclear or potentially ambiguous wording. Based on the review and preliminary check, wording was refined where necessary before administration. In addition, all measures were administered using a five-point response structure to reduce variation in the number of response options across the questionnaire. The adequacy of the adapted Arabic measures was subsequently examined in the reflective measurement model through item loadings, reliability coefficients, convergent validity, and discriminant validity. These procedures were intended to support linguistic clarity and preservation of the intended item meaning in the Arabic versions and were informed by general recommendations for test translation and adaptation (International Test Commission, 2017).

#### 2.3.1 Academic social comparison orientation

Academic social comparison orientation was assessed using an academic adaptation of the Arabic INCOM short form developed and validated by Almailabi and Buunk (2026). The Arabic INCOM short form consists of seven items rated on a five-point Likert scale ranging from 1 (strongly disagree) to 5 (strongly agree) and contains no reverse-worded items. The original short form covers two content domains: ability (4 items) and opinion (3 items). For the purposes of the present study, the item wording was adapted to the academic context so that the items referred specifically to students’ comparison of their academic abilities and academic opinions with those of their peers. ASCO was modeled as a single reflective construct using all seven indicators because the substantive focus of the study was on students’ overall academic comparison orientation rather than on separate ability- and opinion-based comparison processes. Higher scores indicate a greater tendency toward academic social comparison orientation.

#### 2.3.2 Academic anxiety

Academic anxiety was assessed using the Academic Anxiety Scale (AAS; Cassady et al., 2019; Finch et al., 2024). The AAS is a unidimensional self-report measure designed to assess generalized academic anxiety across educational settings. It consists of 11 items rated on a five-point Likert-type scale ranging from 1 (not at all typical of me) to 5 (very typical of me). Higher scores indicate greater academic anxiety.

#### 2.3.3 Academic self-efficacy

Academic self-efficacy was assessed using the General Academic Self-Efficacy Scale (GASE; Nielsen et al., 2018). The GASE is a brief self-report measure consisting of five items designed to assess students’ beliefs about their ability to deal effectively with academic demands. Responses are rated on a five-point Likert scale ranging from 1 (strongly disagree) to 5 (strongly agree). Higher scores indicate higher levels of academic self-efficacy.

#### 2.3.4 Academic help-seeking

Adaptive academic help-seeking was assessed using an adapted version of the adaptive help-seeking dimension developed by Ryan and Pintrich (1997). In its original formulation, this dimension assesses students’ tendency to seek help in ways that support independent understanding and continued task engagement rather than relying on others to provide complete solutions. The adapted measure used in the present study consisted of five items rated on a five-point Likert-type scale ranging from 1 (strongly disagree) to 5 (strongly agree). For the purposes of the present study, the item wording was adapted to reflect the general academic context of university study. Higher scores indicate a greater tendency toward adaptive academic help-seeking.

### 2.4 Common method bias

Because the study relied on self-reported questionnaire data collected from a single source in a cross-sectional design, common method bias was considered a potential methodological concern. Following the procedural recommendations outlined by Podsakoff et al. (2003), several ex ante precautions were implemented to reduce this risk, including informing participants that their responses would be treated as confidential, avoiding the collection of direct identifying information, emphasizing the voluntary nature of participation, and clarifying that responses would be used for research purposes only. In addition, because common method bias in PLS-SEM may increase collinearity among latent variables and thereby bias path estimates, it was assessed statistically using the full collinearity approach recommended by Kock (2015). Specifically, full collinearity variance inflation factor (VIF) values were examined for all latent constructs, with values exceeding 3.3 taken to indicate potential common method bias. In the present study, this approach was used as the primary statistical diagnostic, whereas *post hoc* marker-based and unmeasured latent method construct procedures were not adopted as the main *post hoc* corrective approach, given that evidence regarding their effectiveness remains mixed and, in some cases, limited (Richardson et al., 2009).

### 2.5 Data analysis strategy

The data were analyzed using partial least squares structural equation modeling (PLS-SEM) in SmartPLS 4.4.1.8. PLS-SEM was considered appropriate because the analytical objective combined examination of the hypothesized structural associations with assessment of the explained variance and predictive relevance of the endogenous constructs. This prediction-oriented component is consistent with methodological guidance identifying the testing of theoretical frameworks from a prediction perspective as a relevant consideration when using PLS-SEM (Hair et al., 2019).

A sensitivity power analysis was conducted using G*Power 3.1 (Faul et al., 2009). For the most complex endogenous construct, using an F-test for linear multiple regression with R^2^ increase, α = 0.05, power = 0.80, *N* = 218, one tested predictor, and four total predictors, the sample was sufficient to detect incremental effects of approximately f^2^ = 0.036 or larger with 80% power. This value falls within Cohen’s (1988) conventional small-effect range for f^2^; therefore, smaller effects may have been underpowered. Because all constructs were specified as reflective, the analysis proceeded in two stages: evaluation of the measurement model followed by evaluation of the structural model. The measurement model was assessed using standardized outer loadings, Cronbach’s alpha, rho_A, composite reliability (rho_C), average variance extracted (AVE), the Fornell–Larcker criterion, and the heterotrait–monotrait ratio of correlations (HTMT). Following established guidelines for reflective measurement models, outer loadings of approximately 0.70 or higher were preferred. Indicators with loadings between 0.40 and 0.70 were considered for retention or removal based on their contribution to composite reliability, AVE, and content validity (Hair et al., 2019, 2022). Cronbach’s alpha, rho_A, and rho_C values of 0.70 or higher, and AVE values of 0.50 or higher, were used as benchmarks for internal consistency reliability and convergent validity, respectively. Discriminant validity was assessed using the Fornell–Larcker criterion and HTMT, with HTMT values interpreted primarily against the 0.85 criterion and 0.90 considered for conceptually similar constructs (Henseler et al., 2015).

The structural model was evaluated by examining inner variance inflation factor (VIF) values, path coefficients, bootstrapped confidence intervals, t values, *p* values, coefficients of determination (R^2^), adjusted R^2^, and effect sizes (f^2^). VIF values below 3 were preferred, although values below 5 were considered acceptable. In interpreting structural effects, f^2^ values of 0.02, 0.15, and 0.35 were considered indicative of small, medium, and large effects, respectively (Hair et al., 2019, 2022). The direct, indirect, and interaction effects in the PLS-SEM model were tested using bootstrapping with 5,000 resamples.

Predictive relevance was assessed using PLSpredict based on Q^2^_predict values and prediction-error indices. Because a full comparison with a linear model benchmark was not reported, the predictive results were interpreted as evidence of predictive relevance rather than high predictive power.

## 3 Results

### 3.1 Sample characteristics

The final analytic sample consisted of 218 undergraduate students. Participants had a mean age of 22.20 years (SD = 1.20). Table 1 presents the demographic characteristics of the final analytic sample, including university, gender, university year, field of study, and academic performance.

**Table 1:** Demographic characteristics of the final analytic sample (*N* = 218).

Variable	Category	Frequency (*n*)	Percentage (%)
University	Islamic University of Madinah	128	58.7
	Taibah University	90	41.3
Gender	Male	117	53.7
	Female	101	46.3
University year	First year	62	28.4
	Second year	46	21.1
	Third year	52	23.9
	Fourth year	58	26.6
Field of study	Theoretical track	120	55.0
	Scientific track	98	45.0
Academic Performance (GPA)	Excellent (4.50–5.00)	40	18.3
	Very Good (3.75–4.49)	87	39.9
	Good (2.75–3.74)	73	33.5
	Below 2.75	18	8.3

### 3.2 Descriptive statistics

Descriptive statistics for the study variables are presented in Table 2. Because all measures were administered using a five-point response structure, summed scores were also converted into item-level means to support interpretation. The item-level means were close to the midpoint of the response scale for academic social comparison orientation (M = 3.00), academic anxiety (M = 3.15), academic self-efficacy (M = 3.03), and adaptive academic help-seeking (M = 3.04), suggesting moderate average levels of the study constructs. Skewness and kurtosis values were within commonly acceptable ranges, indicating no substantial departures from distributional symmetry in the summed scale scores.

**Table 2:** Descriptive statistics for the study variables.

Construct	*N*	M	SD	Skewness	Kurtosis
ASCO	218	21.00	6.00	0.060	−0.542
AA	218	34.61	9.23	0.055	−0.823
ASE	218	15.17	4.13	0.062	−0.648
AHS	218	15.22	4.07	−0.154	−0.380

ASCO, academic social comparison orientation; AA, academic anxiety; ASE, academic self-efficacy; AHS, adaptive academic help-seeking. Descriptive statistics were computed using summed scale scores.

### 3.3 Common method bias diagnostic

Common method bias was examined using the full collinearity variance inflation factor (VIF) approach. The full collinearity VIF values were 1.188 for academic social comparison orientation, 1.183 for academic anxiety, 1.136 for academic self-efficacy, and 1.200 for adaptive academic help-seeking. All values were below the 3.3 threshold recommended by Kock (2015). Accordingly, the full collinearity results did not indicate a serious common method bias concern under this diagnostic criterion.

### 3.4 Assessment of the reflective measurement model

The reflective measurement model was assessed before interpreting the structural relationships. Following established PLS-SEM guidelines, indicator reliability, internal consistency reliability, and convergent validity were evaluated using standardized outer loadings, Cronbach’s alpha, rho_A, composite reliability (rho_C), and average variance extracted (AVE) (Hair et al., 2019, 2022). As shown in Figure 2, most standardized outer loadings met or exceeded the recommended benchmark of approximately 0.70. Three indicators were marginally below this benchmark: AA1 (0.686), ASE3 (0.696), and AHS3 (0.686). These indicators were retained because their loadings were close to 0.70 and the corresponding construct-level reliability and convergent validity indices remained within acceptable ranges. Consistent with the decision rule specified in the Data Analysis Strategy, indicator deletion was therefore not considered warranted solely because these loadings fell slightly below the approximate 0.70 benchmark (Hair et al., 2019, 2022). As reported in Table 3, Cronbach’s alpha values ranged from 0.811 to 0.913, rho_A values ranged from 0.831 to 0.917, and rho_C values ranged from 0.866 to 0.926, indicating adequate internal consistency reliability. In addition, AVE values ranged from 0.534 to 0.585, exceeding the recommended 0.50 criterion. Overall, the results provide adequate support for the internal consistency reliability and convergent validity of the reflective constructs.

**Figure 2:**
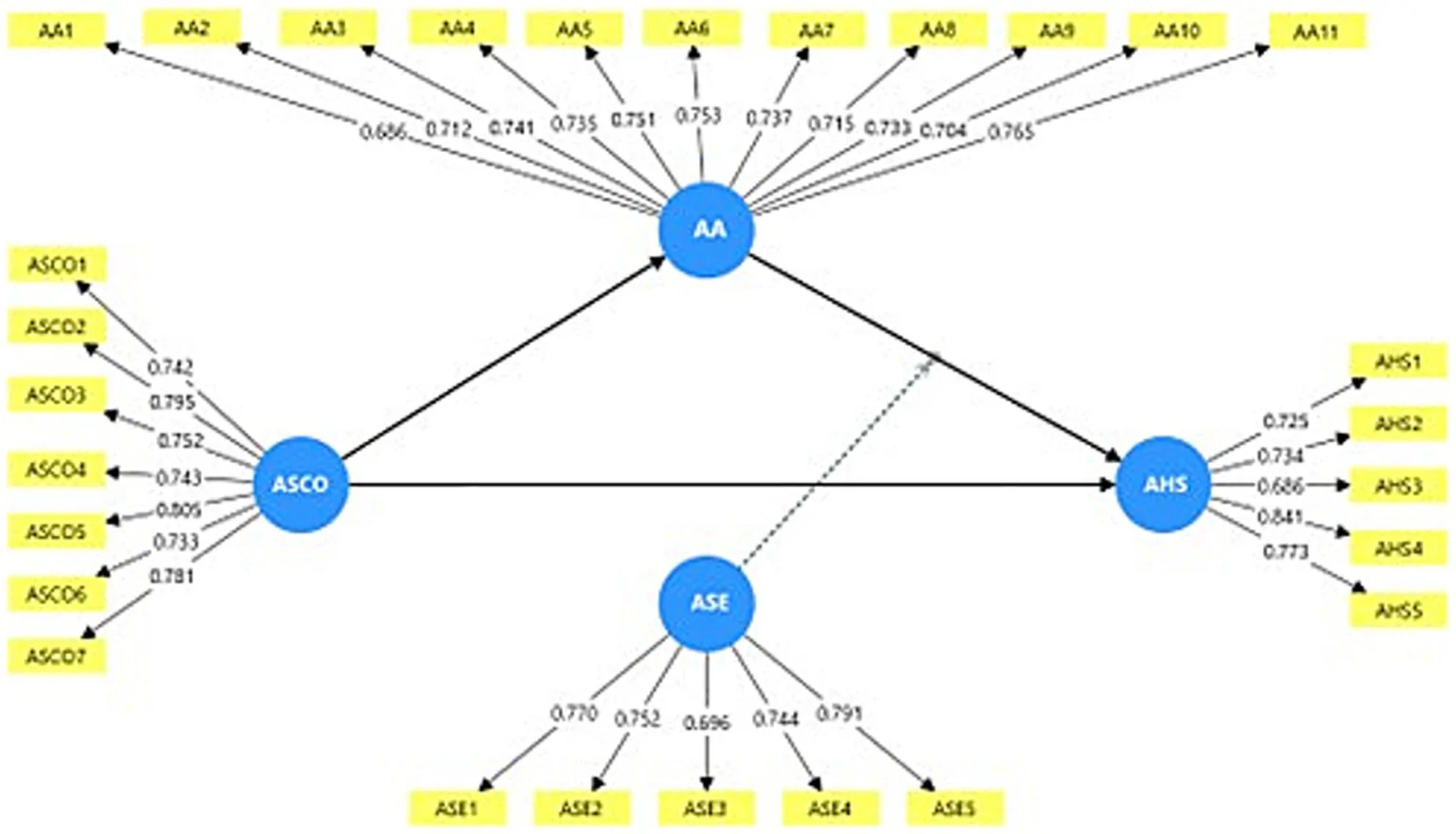
PLS-SEM path model displaying standardized outer loadings for the reflective constructs. ASCO, academic social comparison orientation; AA, academic anxiety; ASE, academic self-efficacy; AHS, adaptive academic help-seeking. The values shown beside the indicator arrows are standardized outer loadings. The dashed arrow represents the moderating effect of academic self-efficacy on the relationship between academic anxiety and adaptive academic help-seeking.

**Table 3:** Indicator loadings, internal consistency reliability, and convergent validity of the reflective constructs.

Construct	No. of items	Loading range	Cronbach’s α	rho_A	rho_C	AVE
AA	11	0.686–0.776	0.913	0.917	0.926	0.534
AHS	5	0.686–0.862	0.812	0.851	0.867	0.568
ASCO	7	0.733–0.810	0.883	0.890	0.908	0.585
ASE	5	0.696–0.843	0.811	0.831	0.866	0.565

ASCO, academic social comparison orientation; AA, academic anxiety; ASE, academic self-efficacy; AHS, adaptive academic help-seeking; rho_A, Dijkstra–Henseler’s reliability coefficient; rho_C, composite reliability; AVE, average variance extracted.

#### 3.4.1 Discriminant validity

Discriminant validity was examined using the Fornell–Larcker criterion and the heterotrait–monotrait ratio of correlations (HTMT). As shown in Table 4, the square root of the AVE for each reflective construct was greater than its absolute correlations with the other constructs. The diagonal values ranged from 0.731 to 0.765 and exceeded the corresponding interconstruct correlations, the largest of which was 0.374. These results were consistent with discriminant validity according to the Fornell–Larcker criterion. In addition, as reported in Table 5, all HTMT values were below the conservative 0.85 threshold commonly used for assessing discriminant validity (Henseler et al., 2015). The highest HTMT value was 0.394, observed between academic social comparison orientation and academic anxiety. These findings provide adequate support for discriminant validity in the measurement model.

**Table 4:** Fornell–Larcker criterion.

Construct	AA	AHS	ASCO	ASE
AA	**0.731**			
AHS	−0.253	**0.753**		
ASCO	0.374	−0.181	**0.765**	
ASE	0.048	0.266	0.203	**0.751**

AA, academic anxiety; ASE, academic self-efficacy; AHS, adaptive academic help-seeking; ASCO, academic social comparison orientation. Bold diagonal values represent the square root of the average variance extracted (AVE) for each construct.

**Table 5:** Heterotrait–monotrait ratio of correlations (HTMT).

Construct	AA	AHS	ASCO
AA			
AHS	0.273		
ASCO	0.394	0.217	
ASE	0.113	0.306	0.252

AA, academic anxiety; ASE, academic self-efficacy; AHS, adaptive academic help-seeking; ASCO, academic social comparison orientation.

### 3.5 Assessment of the structural model

After establishing the adequacy of the measurement model, the structural model was assessed by examining collinearity, explained variance, and effect sizes. As shown in Table 6, the inner VIF values ranged from 1.000 to 1.236, indicating that collinearity among the predictor constructs was not a concern. The model explained 14.0% of the variance in academic anxiety (R^2^ = 0.140, adjusted R^2^ = 0.136) and 18.9% of the variance in adaptive academic help-seeking (R^2^ = 0.189, adjusted R^2^ = 0.174). The f^2^ values ranged from 0.030 to 0.162. Most effects were small according to conventional guidelines, whereas the effect of academic social comparison orientation on academic anxiety was slightly above the conventional medium-effect benchmark (f^2^ = 0.162). The next largest effect was observed for academic self-efficacy on adaptive academic help-seeking (f^2^ = 0.123). Overall, these results indicated acceptable collinearity diagnostics and modest explanatory power for the structural model.

**Table 6:** Structural collinearity statistics, explained variance, and effect sizes.

Criterion	Path / Endogenous construct	Value
VIF	ASCO → AA	1.000
VIF	AA → AHS	1.195
VIF	ASCO → AHS	1.236
VIF	ASE → AHS	1.052
VIF	ASE × AA → AHS	1.080
R^2^	AA	0.140
Adjusted R^2^	AA	0.136
R^2^	AHS	0.189
Adjusted R^2^	AHS	0.174
f^2^	ASCO → AA	0.162
f^2^	AA → AHS	0.056
f^2^	ASCO → AHS	0.037
f^2^	ASE → AHS	0.123
f^2^	ASE × AA → AHS	0.030

### 3.6 Hypothesis testing

The direct and interaction effects in the structural model are reported in Table 7 and are interpreted below in relation to the study hypotheses.

**Table 7:** Direct and interaction effects in the structural model.

Path	β	M	SD	t	*p*	95% CI	Result
ASCO → AA	0.374	0.386	0.058	6.447	<0.001	[0.270, 0.496]	Supported
AA → AHS	−0.234	−0.238	0.072	3.248	0.001	[−0.376, −0.094]	Supported
ASE → AHS	0.324	0.339	0.061	5.309	<0.001	[0.219, 0.457]	Significant main effect
ASCO → AHS	−0.192	−0.194	0.067	2.871	0.004	[−0.327, −0.062]	Significant
ASE × AA → AHS	0.153	0.148	0.059	2.592	0.010	[0.031, 0.263]	Supported

ASCO, academic social comparison orientation; AA, academic anxiety; ASE, academic self-efficacy; AHS, adaptive academic help-seeking; β, standardized path coefficient; M, bootstrap sample mean; SD, standard deviation; CI, confidence interval.

#### 3.6.1 H1: academic social comparison orientation and academic anxiety

H1 proposed that academic social comparison orientation would be positively associated with academic anxiety. As reported in Table 7, academic social comparison orientation was positively associated with academic anxiety (β = 0.374, t = 6.447, *p* < 0.001, 95% CI [0.270, 0.496]). Thus, H1 was supported.

#### 3.6.2 H2: academic anxiety and adaptive academic help-seeking

H2 proposed that academic anxiety would be negatively associated with adaptive academic help-seeking. As reported in Table 7, academic anxiety was negatively associated with adaptive academic help-seeking (β = −0.234, t = 3.248, *p* = 0.001, 95% CI [−0.376, −0.094]). Thus, H2 was supported.

#### 3.6.3 H3: statistical mediation through academic anxiety

H3 proposed that academic anxiety would statistically mediate the association between academic social comparison orientation and adaptive academic help-seeking. The mediating effect of academic anxiety was examined using the bootstrapped specific indirect effect. As shown in Table 8, the indirect effect of academic social comparison orientation on adaptive academic help-seeking through academic anxiety was negative and statistically significant (β = −0.087, t = 2.812, *p* = 0.005, 95% CI [−0.156, −0.034]). Because the confidence interval did not include zero, H3 was supported. In addition, the direct effect of academic social comparison orientation on adaptive academic help-seeking was statistically significant in the model including academic anxiety (β = −0.192, *p* = 0.004), consistent with partial statistical mediation in the estimated model. Thus, higher academic social comparison orientation was associated with higher academic anxiety, which in turn was associated with lower adaptive academic help-seeking.

**Table 8:** Specific indirect effect.

Indirect path	β	M	SD	t	p	95% CI	Result
ASCO → AA → AHS	−0.087	−0.092	0.031	2.812	0.005	[−0.156, −0.034]	Supported

ASCO, academic social comparison orientation; AA, academic anxiety; AHS, adaptive academic help-seeking; β, standardized indirect effect; CI, confidence interval.

#### 3.6.4 H4: moderating role of academic self-efficacy

H4 proposed that academic self-efficacy would moderate the association between academic anxiety and adaptive academic help-seeking, such that the negative association would be weaker at higher levels of academic self-efficacy. As reported in Table 7, the interaction effect in the PLS-SEM model was positive and statistically significant (β = 0.153, t = 2.592, *p* = 0.010, 95% CI [0.031, 0.263]), supporting H4. Academic self-efficacy also showed a positive main effect on adaptive academic help-seeking (β = 0.324, t = 5.309, *p* < 0.001, 95% CI [0.219, 0.457]). The positive interaction coefficient indicates that the negative association between academic anxiety and adaptive academic help-seeking became weaker as academic self-efficacy increased.

### 3.7 Summary of hypothesis testing

Table 9 summarizes the results for H1–H4.

**Table 9:** Summary of hypothesis testing.

Hypothesis/effect	Relationship	*β* / Effect	*p* / 95% CI	Result
H1	ASCO → AA	0.374	*p* < 0.001; 95% CI [0.270, 0.496]	Supported
H2	AA → AHS	−0.234	*p* = 0.001; 95% CI [−0.376, −0.094]	Supported
H3: Mediation	ASCO → AA → AHS	−0.087	*p* = 0.005; 95% CI [−0.156, −0.034]	Supported
H4: Moderation	ASE × AA → AHS	0.153	*p* = 0.010; 95% CI [0.031, 0.263]	Supported

ASCO, academic social comparison orientation; AA, academic anxiety; ASE, academic self-efficacy; AHS, adaptive academic help-seeking.

### 3.8 Supplementary analyses

#### 3.8.1 Predictive relevance

The model’s predictive relevance was assessed using PLSpredict. As shown in Table 10, the Q^2^_predict values were positive for both endogenous constructs: academic anxiety (Q^2^_predict = 0.120) and adaptive academic help-seeking (Q^2^_predict = 0.093). These results provide initial evidence of predictive relevance for the endogenous constructs (Shmueli et al., 2019). Because the present reporting is based primarily on Q^2^_predict values and does not include a full comparison with a linear model benchmark, the findings should be interpreted conservatively as evidence of predictive relevance rather than evidence of high predictive power.

**Table 10:** PLSpredict results for endogenous constructs.

Endogenous construct	Q^2^_predict	RMSE	MAE
AA	0.120	0.945	0.791
AHS	0.093	0.963	0.765

RMSE, root mean squared error; MAE, mean absolute error. Positive Q^2^, predict values indicate predictive relevance relative to a naïve benchmark. The table does not assess the degree of predictive power against a linear model benchmark.

## 4 Discussion

The present study examined academic anxiety as a mediator and academic self-efficacy as a moderator in the association between academic social comparison orientation and adaptive academic help-seeking among university students. Overall, the findings were consistent with the main structure of the proposed model. Academic social comparison orientation was positively associated with academic anxiety, and academic anxiety was negatively associated with adaptive academic help-seeking. The indirect effect through academic anxiety was statistically significant, consistent with partial statistical mediation between academic social comparison orientation and adaptive academic help-seeking, whereas academic self-efficacy statistically moderated the negative association between academic anxiety and adaptive academic help-seeking, such that this association became weaker as academic self-efficacy increased. Because the study was cross-sectional, these findings should be interpreted as theoretically informed associations rather than as evidence of causal pathways. The findings suggest that peer-referenced academic self-evaluations, academic anxiety, and academic self-efficacy are jointly relevant to understanding students’ self-reported adaptive academic help-seeking.

### 4.1 Academic social comparison orientation and academic anxiety

The finding that academic social comparison orientation was positively associated with academic anxiety suggests that students who reported a stronger tendency to compare their academic abilities and opinions with those of their peers tended to report higher levels of academic anxiety. This interpretation follows from the way the construct was operationalized: the academically adapted INCOM assessed a greater tendency to engage in academic social comparison orientation rather than students’ perceived superiority or inferiority relative to peers. In this sense, the results indicate that the tendency to use peers as academic comparison referents was associated with greater anxiety in academic settings.

This result fits broader evidence showing that social comparison is associated with anxiety-related outcomes (McCarthy and Morina, 2020), as well as experience-sampling evidence indicating that social comparisons in daily life are relevant to anxiety-related experiences among undergraduate participants (Goodman et al., 2021). One plausible explanation is that students with stronger academic comparison orientation may evaluate academic tasks, performance, and competence more through peer-referenced standards, making academic situations more evaluatively salient and therefore more closely tied to anxiety. However, this study did not assess the direction or content of comparison, such as whether students engaged mainly in upward, downward, favorable, or unfavorable comparisons. Therefore, the result should be understood as evidence that academic social comparison orientation is associated with academic anxiety, not as evidence that a specific type of comparison is responsible for this association.

### 4.2 Academic anxiety and adaptive academic help-seeking

The negative association between academic anxiety and academic help-seeking indicates that students who reported higher academic anxiety tended to report lower levels of adaptive academic help-seeking. This result aligns with the conceptualization of academic help-seeking as self-regulatory academic behavior rather than a simple reaction to difficulty. Li et al. (2023) described academic help-seeking as an important self-regulation strategy for college students, whereas Ryan and Pintrich (1997) emphasized that help-seeking involves recognizing the need for assistance and deciding whether and how to obtain help from others.

Because help-seeking commonly involves interaction with others, it is also shaped by the social meaning of requesting assistance. Karabenick (2004) noted that help-seeking is inherently social and therefore more sensitive to contextual and interpersonal features than many other learning strategies. From this perspective, academic anxiety may be linked to lower adaptive help-seeking when students with higher academic anxiety experience the act of requesting assistance as more evaluative or threatening than as a normal learning strategy. This interpretation is compatible with Ryan and Pintrich’s (1997) distinction between adaptive help-seeking and help-seeking avoidance, particularly their finding that perceived threats and benefits were important for understanding avoidance of help-seeking, whereas perceived benefits predicted adaptive help-seeking.

The result also corresponds with Federici et al. (2015), who reported that, in mathematics classrooms, mastery orientation was associated with lower anxiety and greater help-seeking, whereas performance-avoidance orientation was linked to higher anxiety and less help-seeking. Because Federici et al.’s evidence came from middle-school mathematics classrooms, the present finding should be interpreted cautiously as convergent evidence from a broader university context. Overall, the results suggest that academic anxiety is associated with lower adaptive help-seeking, possibly reflecting greater salience of the social and evaluative aspects of requesting assistance among students reporting higher academic anxiety.

### 4.3 Mediating role of academic anxiety

The mediation analysis integrated the preceding direct associations by showing that academic anxiety partially mediated the association between academic social comparison orientation and adaptive academic help-seeking. This result indicates that students’ tendency to compare their academic abilities and opinions with those of their peers was associated with lower adaptive help-seeking, partly through higher academic anxiety. Thus, academic anxiety is best treated as **a** statistical intermediate in the tested model.

A theoretically plausible account of this indirect pattern integrates the mechanisms discussed in the preceding sections. Stronger academic comparison orientation may heighten the salience of peer-referenced evaluation and correspond with greater academic anxiety, while heightened evaluative concern may make requesting assistance feel more threatening or costly. Taken together, these processes provide one plausible account of why stronger academic comparison orientation was indirectly associated with lower adaptive help-seeking through higher academic anxiety.

Methodologically, this conclusion rests on the significant bootstrapped indirect effect, which provides evidence of partial statistical mediation in the estimated model. However, because the study used a cross-sectional design, the mediation should not be interpreted as evidence of temporal ordering or a causal sequence. The remaining significant direct effect from academic social comparison orientation to adaptive academic help-seeking further indicates that academic anxiety was statistically linked to part, but not all, of this association.

### 4.4 Moderating role of academic self-efficacy

The moderation analysis showed that academic self-efficacy statistically moderated the negative association between academic anxiety and adaptive academic help-seeking. The positive interaction coefficient indicated that this negative association became weaker as academic self-efficacy increased. This finding suggests that students with stronger academic self-efficacy may be less likely to show reduced adaptive help-seeking when they report higher academic anxiety.

Conceptually, this interpretation is consistent with the social-cognitive view of self-efficacy as a context-specific belief that shapes motivation, self-regulation, effort, and emotional responses in academic settings (Pajares, 1996). When students believe that they can manage academic demands, they may be more likely to interpret difficulty as manageable and to view help-seeking as an instrumental learning strategy rather than as evidence of incompetence. Help-seeking research likewise indicates that requesting assistance is both a self-regulatory strategy and a socially embedded and potentially evaluative behavior, with perceived threat being especially relevant to help-seeking avoidance (Ryan and Pintrich, 1997).

In this sense, academic self-efficacy may help characterize conditions under which academic anxiety is less strongly associated with reduced help-seeking. Related evidence from undergraduate samples has linked academic self-efficacy to students’ academic help-seeking behaviors (Xie and Xie, 2019). Yang et al. (2025) similarly found, across one questionnaire study and three experimental vignette studies, that academic self-efficacy buffered the negative effect of help-seeking threat on students’ help-seeking behaviors, although their study focused on middle-school students and help-seeking threat rather than university students’ academic anxiety. Taken together, these findings are compatible with a buffering pattern associated with academic self-efficacy in the anxiety–help-seeking association.

### 4.5 Practical implications

The findings suggest that university support practices may benefit from addressing both the evaluative and self-regulatory dimensions of academic help-seeking. Because academic social comparison orientation was positively associated with academic anxiety, instructors and academic advisors may need to be cautious about learning environments that make peer comparison highly salient without emphasizing mastery, improvement, and available support.

The results also suggest that efforts to normalize help-seeking as a legitimate learning strategy may be useful, rather than framing it, implicitly or explicitly, as evidence of academic weakness. This implication is particularly relevant because academic anxiety was associated with lower adaptive academic help-seeking and statistically mediated the association between academic social comparison orientation and adaptive academic help-seeking. Finally, because the negative association between academic anxiety and adaptive help-seeking was weaker at higher levels of academic self-efficacy, academic support programs may benefit from strengthening students’ confidence in managing academic demands through structured feedback, scaffolded tasks, academic skills training, and accessible advising. These implications should be viewed as targeted, theory-informed recommendations rather than comprehensive intervention claims, given the cross-sectional design and the modest explanatory power of the model.

### 4.6 Limitations and future directions

The study design places an important boundary on interpretation. Because the data were cross-sectional, the mediation involving academic anxiety cannot establish temporal ordering or causality. The mediation and moderation findings are therefore best treated as statistical patterns within the tested model. Longitudinal, cross-lagged, or experimental work is needed to examine whether academic social comparison orientation precedes changes in academic anxiety and adaptive academic help-seeking, and whether academic self-efficacy weakens these associations over time.

Another limitation concerns the use of single-source self-reported data. Although procedural precautions and full collinearity diagnostics were used to address common method bias, this source of bias cannot be ruled out entirely. Evidence from behavioral indicators of adaptive academic help-seeking, learning management system records, or reports from peers, instructors, or advisors would provide a stronger basis for evaluating the model.

The measurement approach also requires further consideration. Although the adapted Arabic measures showed acceptable reliability, convergent validity, and discriminant validity indices in the present sample, some instruments were translated, contextually adapted, or administered using a five-point response structure. Additional work is needed to examine the measurement properties of these adapted forms across different university samples.

The sample was drawn from undergraduate students at two Saudi universities, which limits the generalizability of the findings beyond similar higher education contexts. The voluntary, nonprobability sampling approach further limits claims regarding the representativeness of the sample. Testing the model across different institutions, academic disciplines, and cultural settings would clarify how context-dependent the observed associations are.

The academically adapted INCOM assesses students’ general tendency to compare their academic abilities and opinions with peers, but it does not assess the direction or content of comparison. Distinguishing upward, downward, favorable, and unfavorable academic comparisons would provide a more precise account of which forms of comparison are most relevant to academic anxiety and help-seeking.

Finally, the study focused on adaptive academic help-seeking. Extending the model to help-seeking avoidance and expedient help-seeking would help determine whether similar associations apply to different forms of help-seeking.

## Data Availability

The raw data supporting the conclusions of this article will be made available by the authors, without undue reservation.
